# Multimodal Behavior Program for ADHD Incorporating Yoga and Implemented by High School Volunteers: A Pilot Study

**DOI:** 10.5402/2011/780745

**Published:** 2011-08-11

**Authors:** Sanjiv Mehta, Vijay Mehta, Sagar Mehta, Devesh Shah, Ashok Motiwala, Jay Vardhan, Naina Mehta, Devendra Mehta

**Affiliations:** ^1^Columbia College, Columbia University, New York, NY 10027, USA; ^2^Department of Chemistry, New College of Florida, Sarasota, FL 34243, USA; ^3^Trinity College of Arts and Science, Duke University, Durham, NC 27708, USA; ^4^UCL Medical School, University College London, London WC1 E6BT, UK; ^5^Harsh Vardhan Memorial Charitable Trust, Safdarjung Enclave, New Delhi, India; ^6^MDKV Inter College, Uttar Pradesh, Najibabad 246763, India; ^7^Arnold Palmer Hospital for Children, Orlando, FL 32806, USA

## Abstract

A low-cost resource approach to ADHD therapy would be a practical approach to treating children in developing countries. Research has shown that ADHD is prevalent in all areas of the world, and yet treatment for children in more impoverished countries is still lacking. The approach taken was to combine yoga and meditation combined with multimodal behavioral therapy program for children ageing 6 to 11. The program was kept low cost by using trained high school volunteers and integrating the program within the public school. After 6 weeks of the program, 90.5% of children showed improvement as measured by their performance impairment score, a measurement of academic performance. Parent and Teacher evaluations of behavior also found improvement as 25 of the 64 children (39.1%) improved into the normal range as measured by the Vanderbilt questionnaire. Moreover, children could successfully learn both yoga and meditation from high school students irrespective of their age, ADHD type, or initial performance impairment. The results demonstrate efficacy of a multimodal behavioral program incorporating yoga and meditation. The use of high school volunteers from schools in the area demonstrates an effective low-cost and universally applicable approach.

## 1. Introduction

Attention Deficit Hyperactivity Disorder (ADHD) is a common disorder that affects 5.3% to 20% of the children worldwide [1]. US studies have shown a prevalence of 8.7% in 8–15 years old (Froehlich et al. 2007) [2]. Specifically in India, studies in hospital or outpatient clinics, with referral bias, suggest prevalence of 5.2% to 29.5% [3–6]. The condition generally leads to poor academic performance and problems with behavior at home and school. Children with this disorder often have other problems such as anxiety, depression, and learning disabilities. As they reach adolescence, these children are also at greater risk of drug and alcohol abuse and other issues such as increased rate of motor vehicle accidents. Children with ADHD also suffer from higher levels of temper-tantrums, tics, and problems with family and peer relationships. If the condition remains untreated, it can continue into adulthood and prevent the person from achieving their maximum potential [7].

With proper medical attention and care, children can generally learn to cope with their disorder. Both medication and behavioral therapy may help. Current treatment involves a multimodal approach that includes medicine along with behavioral therapy. Drugs, which usually consist of stimulants such as methylphenidate and amphetamine-dextroamphetamine, are either expensive or not available in India. Furthermore, these medications require close medical attention, which is also a scarce and expensive resource. Indeed, while medication combined with behavioral therapy has been shown to be the most effective therapy after 1 year and 2 years in the Multimodal Treatment Study of Children with Attention Deficit Hyperactivity Disorder conducted by NIMH, longer term followup suggests medications appear to have little additional benefit [8]. Unfortunately, there is a lack of similar studies in India regarding the use of stimulant medication, or multimodal therapy.

Clearly, the use of a low-cost, effective method for identifying children with ADHD and providing them with some form of behavioral therapy would go a long way to improve the quality of life for a significant portion of the school age population. Some approaches, such as the use of play therapy and physical exercise, would be easy to adapt. Ideally, strategies that are culturally familiar would be more acceptable and easier to incorporate. 

We devised a program combining yoga, meditation, and play therapy. There is strong belief but limited evidence to show that yoga and meditation help focus and attention. Pilot studies using this as family-based therapy for ADHD in 8 boys have been reported to show promise [9]. Additional theoretical basis may be the increase in dopamine release in the CNS from yoga [10]. In children with ADHD, reduced dopamine levels are seen in the CNS, and strategies to regulate these levels are suggested as possible therapies [11].

 To make this affordable, a peer-mediated approach utilizing normal high school volunteers was planned. Indeed, limited studies in small settings have shown peer tutoring does help children with ADHD [12, 13]. Furthermore, to allow maximum adherence, immersion in the regular school day was planned. 

We report efficacy of a six-week multimodal peer-mediated behavioral program that includes yoga in improving performance and behavioral scores as measured by the Vanderbilt. Additionally, the ability of peers to instruct and children with ADHD to learn yoga was evaluated. Finally, whether this embedded program could remain functional based solely on local resources was also assessed.

## 2. Methods

### 2.1. Population

A program was designed to assess prevalence of ADHD in India and perform a needs assessment in the town of Najibabad 250 kilometers north of Delhi. The boys and girls schools in this town of 150,000 in North UP accounted for more than 40% of the town's children, from all socioeconomic backgrounds. The majority of children came from urban or semiurban areas (87.2%), and 56% were Hindus while 42% Muslim. All 910 children ageing from 6 to 11 were screened for ADHD using the Initial Teacher Vanderbilt Assessment [6, 7].

### 2.2. Identification

The Vanderbilt questionnaires were translated into Hindi by trained teachers. While it was designed in the English language and is used prevalently in Western countries, this score has been used in other non-English speaking countries [14]. Furthermore, studies analyzing diagnosis of ADHD in children in India using the Vanderbilt have been performed. Malhi et al. found that the Vanderbilt could be used to identify children with ADHD, though some minor discrepancies between parents and teachers reporting information about the child were noted [15]. The performance impairment scores on the questionnaire are based on 8 areas: reading, mathematics, written expression, relationship with peers, following directions, disrupting class, assignment completion, and organizational skills. Each category is scored from a 1 to 5 with a 4 or 5 indicating impairment, and hence abnormal scores. Those with impairment had the parent Vanderbilt questionnaire completed. From this initial screen, 156 with poor school performance were identified, and the combined parent and teacher scores as well as a neurodevelopmental assessment by neurodevelopmental pediatrician identified 80 children (8.8%) diagnosed with ADHD and further categorized into either combined, predominantly inattentive, or predominantly hyperactive/impulsive types.

### 2.3. Ethics

All of the 80 diagnosed children and their parents had informed consent obtained. The study was approved by the school board of MDKV and registered under clinicaltrials.gov (NCT01012778). These children were evaluated in a medical camp for comorbidities, and additional programs to educate families and teachers on the problems of ADHD were conducted. Furthermore, all yoga postures were designed by designated yoga teachers to ensure that they have no real physical strain beyond normal physical activities.

### 2.4. Design

Of these 80 children, 76 enrolled into the program we titled Climb Up. This was a multimodal program that incorporated yoga postures, meditation program, and behavioral play therapy in 1-hour sessions during the school day. Yoga and meditation were performed for the first 25 minutes. Postures and simple breathing techniques that would be appropriate for children between 6 and 11 years were used. The next 30 minutes were devoted to behavioral therapy and then 5 minutes for discussion about the past sessions or any questions the children might have. The children were organized into manageable groups of 8 to 12 children and also divided by gender to keep with school custom. This resulted in a total of 3 female groups and 5 male groups. Each group had 1-hour sessions twice weekly over a period of 6 weeks. Additional practice was suggested daily at home. Teachers played no direct role in the actual therapy of the children but supervised the classroom, and logged children's behavior using a simple scale. Simple games that reward concentration were used as play therapy.

High school volunteers were selected after being recommended by their teachers for excelling in academics and leadership qualities. A further criterion was that they could take on this extra responsibility without adversely affecting their academic performance. The volunteers were trained and assessed over 3 weeks until they became proficient in yoga, meditation, and peer mentoring during the behavioral therapy. From the fifth week, the high school student volunteers managed the program alone.

In the sixth week of the program, children's yoga performance was measured using a yoga posture score (Table 1). In this system, we identified 5 aspects specific for each of the 8 yoga postures. In each of the 5 aspects of the yoga posture, a child could score from 0 to 2. A score of 0 indicated that the specific aspect of the posture was not recognized and the child did not even make an attempt. A score of 1 indicated that the child attempted that aspect of the posture but performed it inadequately. Finally, a score of 2 indicated that the child recognized that aspect of the posture and performed it at an adequate level. This scoring system was used by 2 observers. Both were trained to reduce interobserver variability. Each child was scored independently by the 2 observers and the mean of the scores used. In order to measure the child's ability to perform breathing portion of meditation, the duration for which they could maintain the humming sound on exhalation was recorded. This was based on the principle that improvement from breathing techniques used in meditation leads to prolonged but controlled exhalation. All children were evaluated in the same week and were measured in three trials using a simple stopwatch. Our measurements in yoga and meditation were compared against a control group of 3 healthy children aged 7–11 who participated in the 6-week program alongside the children with ADHD.

In addition, the children were assessed with a follow-up teacher and parent Vanderbilt. This included performance impairment score by the teachers, allowing comparison from baseline for both the total number of categories still impaired and average performance impairment score, as well as behavioral scores. Using the change in the average performance impairment score, percent improvement was calculated per child.

## 3. Results

Completed teacher and parent Vanderbilt follow-up questionnaires were collected for 70 of the 76 children who joined the program. The collected data contained 26 females and 44 males, a gender ratio similar to that seen in the US [1]. The average age for females was 8.27 SD 1.8, while the average age for males was 8.47 SD 1.3. Furthermore, the parent and teacher Vanderbilt scores were used to subcategorize 70 of the children into combined, hyperactive/impulsive, and inattentive. Of these, 47 of the children (67.1%) were combined, 8 of the children (11.4%) were predominantly hyperactive/impulsive, and finally 15 of the children (21.4%) were predominantly inattentive. 

**Figure 1:**
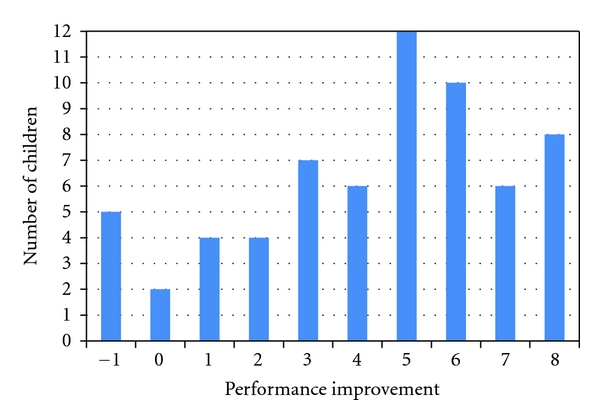
Performance Improvement after 6 weeks. We measured improvement in school performance as rated by the teacher. This shows the distribution of performance improvement in the children after the 6-week program. Overall, 90.5% showed some improvement in these scores.

Follow-up teacher and parent Vanderbilt questionnaires were completed for 63 children. Improvement of performance impairment scores was noted in a large number of children, specifically in the poor academic and social performance categories. The average performance impairment score showed a significant decrease from an average baseline score of 5.72 SD 1.78 to 1.41 SD 2.13 on followup (*P* < 0.001 Paired *t*-Test). More importantly, 57 of the 63 (90.5%) children had some form of improvement in their performance impairment from this therapy, and more than half the students, 35 of the 63 (55.5%) students, improved to the normal range with no performance impairment reported by teachers. The performance impairment score change did not vary by gender, age, or initial ADHD subtype (Figure 1).

Also, final teacher and parent behavioral scores demonstrated an alternative measure of improvement to the performance impairment scores. 25 of the 64 children (39.1%) had behavioral scores shift from the abnormal to normal range as rated by parents and teachers. This improvement also did not depend on age, gender, or ADHD type.

### 3.1. Yoga and Meditation Results

**Figure 2:**
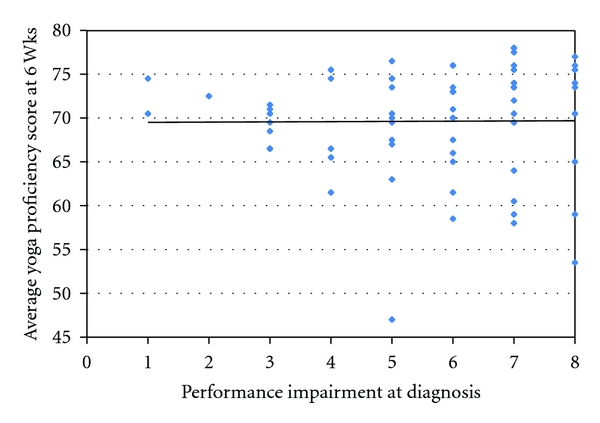
Performance impairment at diagnosis and yoga score. We compared the children's ability to learn yoga with their initial impairment in academic performance. We show that children could learn yoga irrespective of their initial impairment. The slight trend for lower yoga scores with a higher performance impairment score was not significant (*R* = 0).

Yoga posture scores (YPSs) and meditation values were also collected for the 63 children. Children were measured on their improvement of yoga posture scores from a baseline score of 40. All the children improved by an average of 29.5 points or an improvement of 73.9% SD 15.5. The children had an average final YPS of 69.5 SD 6.25. This was similar to the control group of 3 children also taught yoga and meditation. There was weaker interobserver correlation at *R*  0.81 than during training of observers (*R*  0.97) but may partly be explained by day to day variance as measurements were not performed concurrently. The improvement in yoga posture scores from baseline did not vary by gender, ADHD subtype, religion, or most importantly their performance impairment level (*R* = 0) (Figure 2).

### 3.2. Meditation Results

**Figure 3:**
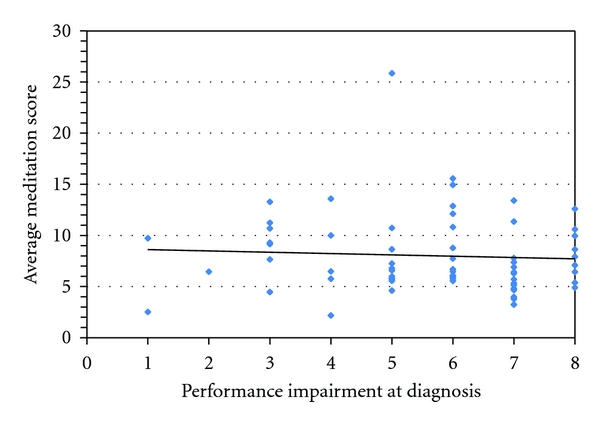
Performance impairment at diagnosis and meditation score. We compared the children's ability to control their breathing with their initial impairment in academic performance. We show that children could learn meditation and control their breath irrespective of their initial impairment score. The slight trend for lower meditation scores with a higher performance impairment score was not significant (*R* = 0.14).

Children could maintain the humming sound/exhaling of breath for an average time of 7.85s SD 3.16 by 6 weeks. Because this had not been performed before, we had no exact expectation for the normal range of child after being taught meditation. We did find, however, that their performance was similar to the control group of 3 children. There was a slight trend for older children to achieve higher meditation scores. Again, this trend did not have statistical significance (*P* = 0.4, ANOVA, *R* = 0.1). Gender and ADHD subtype had no correlation with the children's meditation scores except for a subgroup of girls who were diagnosed with the ADHD combined subtype who had a higher than average meditation score (*P* = 0.06, Chi square). Again, there was no significant correlation between the children's ability to learn meditation and their initial performance impairment score (*R* = 0.14) (Figure 3).

## 4. Discussion

The results of this pilot study demonstrate that a six-week peer-mediated multimodal behavioral program that included Yoga and Meditation can lead to measurable benefits in children with ADHD. More than 50% of the children improved their academic and behavioral performance. Improvement did not really vary by age, gender, or type of diagnosed ADHD. 

The ability to incorporate yoga and meditation as well as play therapy using school aged peers as volunteers was shown. The improvements seen from the program would need to be sustained in the long term, and further prospective studies are needed to dissect out factors that may be relevant to improvement. Furthermore, the fact that the Vanderbilt questionnaire had to be translated into Hindi for all the parents and the majority of the teachers may have led to some misunderstanding and affected some of the results. This questionnaire has been used by others in India but still needs to be formally validated in Hindi. Mahli et al. noted parent-teacher discordance when applied to Indian school children [15]. This potential problem may lead to an error in diagnosis in a few cases but does not affect the main outcomes of the study because the change in performance of each child was analyzed. The difference between parent and teacher would remain consistent and not affect the measured change in performance of the child. 

Through the course of the program, it was evident that the children could and did learn the yoga to a standard level setup by a control group of 3 children. The children learned yoga over the six-week period and improved to an average yoga posture score of 69.5 with an SD 6.25, a score of 86.9%. While this novel score warrants validation and interobserver variation in practice was higher (*R* = 0.81), one promising finding was that the yoga score along with the meditation score fell within a normal distribution curve. The scoring system demonstrated that a majority of the children learned yoga to a similar level. More importantly, Yoga improvement did not vary by gender, age, type of ADHD diagnosed, or performance impairment score at diagnosis. If confirmed, this would suggest therapy would be applicable no matter the variation of age or the severity of the child's ADHD. 

Meditation in terms of deep breathing/humming was also adopted well. The length of controlled exhalation in the majority of the children fell within the average score of 7.85 seconds, similar to a control group of 3 children. Again children could learn to control their breath irrespective of their performance impairment score at diagnosis indicating that their impairment due to ADHD did not inhibit them from learning meditation.

Finally, the program was successfully embedded within the school program. Student volunteers from within the school managed the program independently by week 5. The ability to train peers in a few sessions is an important low-cost strategy. The results of the six-week period show promise of such an approach as an effective and low-cost way to address needs of children with ADHD. Long-term followup of the peer-mediated intervention is ongoing to see if these early gains are sustained.

## Figures and Tables

**Table 1:** Yoga Posture Score Sheet.

File number:			
Name:			
Gender:			

Yoga			

Janushirasana	*x/10*	Hastapadasana	*x/10*
Stretching	x/2	Stretching	x/2
Straight leg	x/2	Leaning back	x/2
Knee down	x/2	Legs straight	x/2
Head to knee	x/2	Hands straight	x/2
Both hands stretching	x/2	Head to knees	x/2

Mountain pose	*x/10*	Tadasana	*x/10*
Stretching	x/2	Stretching	x/2
Arms straight	x/2	Hands interlocked	x/2
Hands together	x/2	Standing on toes	x/2
Butt down	x/2	Hands raised up	x/2
Head to the ground	x/2	Fairly balanced	x/2

Yogmudra	*x/10*	Chakrasana	*x/10*
Stretching	x/2	Stretching	x/2
hands behind back	x/2	Back straight	x/2
leg position	x/2	Arm position	x/2
head to ground	x/2	Legs apart	x/2
arms up	x/2	Legs straight	x/2

Snake pose	*x/10*	Trikonasana	*x/10*
Stretching	x/2	Stretching	x/2
Hand position	x/2	Legs apart	x/2
3 levels	x/2	Arms straight	x/2
Arms bent	x/2	Legs straight	x/2
Feet together	x/2	Arm position	x/2

Meditation			
Time 1			
Time 2			
Time 3			
Average			
